# Balloon Catheter Position and its Relationship with Esophageal Temperature during Pulmonary Vein Isolation using High-Intensity Focused Ultrasound

**DOI:** 10.1016/s0972-6292(16)30542-3

**Published:** 2012-09-01

**Authors:** Kars Neven, Andreas Metzner, Boris Schmidt, Feifan Ouyang, Karl-Heinz Kuck

**Affiliations:** Asklepios Klinik St. Georg, Department of Cardiology, Hamburg, Germany

**Keywords:** Atrial fibrillation, Ablation, Complications, Esophagus, High-intensity focused ultrasound

## Abstract

**Background:**

HIFU can achieve PVI, but severe esophageal complications have happened. We analyzed relative position of HIFU balloon catheter (BC) to esophageal temperature (ET) probe and correlated it to ET changes.

**Methods and Results:**

Before each ablation relative position of HIFU BC to ET probe was recorded in RAO 30º and LAO 40º. We compared ablations where ET at end of ablation was <38.5ºC or ≥38.5ºC and <40.0ºC or ≥40.0ºC.

A total of 600 images from 311 ablations in 28 patients (18 male, age 63±7 years), were analyzed. ET ≥38.5ºC was reached when distance from BC to ET probe was: <20mm in LAO for RSPV and <29mm in LAO for RIPV. For RIPV ET ≥38.5ºC was reached when angle between BC and ET probe was significantly smaller in LAO and RAO. ET ≥40.0ºC was reached when distance of BC to ET probe was: <20mm in LAO for RIPV, <14mm in RAO for RIPV, <18mm in RAO for LIPV. ET increased to ≥40.0ºC when distance from BC to ET probe was significantly longer in LAO for LIPV. For RIPV ET ≥40.0ºC was reached when angle between BC and ET probe was significantly smaller in LAO.

**Conclusion:**

There is a relationship between distance/angle of HIFU BC to ET probe and ET: shorter distances and smaller angles can cause higher ET.

## Introduction

High-intensity focused ultrasound (HIFU) is a balloon-based ablation system for treatment of atrial fibrillation, which does not need balloon-to-wall contact in order to deliver ablation energy effectively.[1] There have been safety issues due to occurrence of severe complications, such as phrenic nerve palsy, pulmonary vein stenosis, stroke and atrial-to-esophageal fistula.[1-3]

Previously, we have reported on 28 patients treated with HIFU in which thermal esophageal damage occurred despite low esophageal temperatures.[4,5] In the present study, from the data collected on these patients we retrospectively investigated the relative position of the HIFU balloon catheter to the esophageal temperature probe and correlated it to changes in esophageal temperature.

## Patients and Methods

### Patient characteristics and Ablation procedure

The patient characteristics, ablation procedure and post-ablation treatment have been described in detail earlier.[4]

### Steerable HIFU balloon catheter

The steerable HIFU balloon catheter consists of a non-compliant balloon, filled with water and contrast medium, and an integrated 9 Megahertz ultrasound crystal. A second non-compliant balloon, filled with carbon dioxide, forms a parabolic surface at the base of the distal balloon. Ultrasound waves are reflected in forward direction, focusing a ring of ultrasound energy (sonicating ring) 2-6 mm to the balloon surface. The balloon catheter is steerable and available in balloons with a sonication ring diameter of 20, 25 and 30 mm. The system is a balloon-based ablation system which does not require balloon-to-wall contact in order to deliver ablation energy effectively. Therefore there is no need for complete occlusion of the pulmonary vein in order to ensure effective energy delivery, unlike cryoballoon and laserballoon systems. The catheter has a central lumen used for insertion of a hexapolar spiral mapping catheter (ProMap™, ProRhythm) for realtime assessment of pulmonary vein potentials.

### Esophageal temperature monitoring

A 3-sensor temperature probe (Esotherm, FIAB, Vicchio, Italy) was advanced in the esophagus. Its position was adjusted for each ablation in order to position the sensors as close as possible to the ablation site, the middle sensor was always placed closest to the balloon.

### Relationship between esophageal temperature and position of the HIFU balloon catheter to the esophageal temperature probe

**Figure 1:**
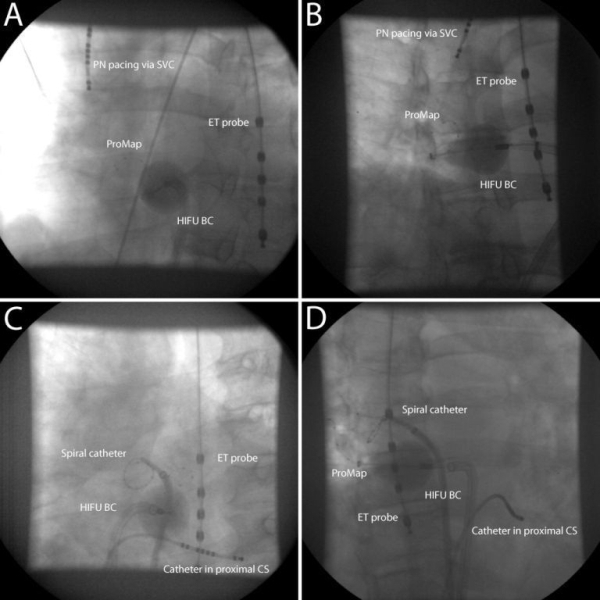
Ablation of RIPV: different positions of HIFU balloon catheter relative to esophageal temperature probe. A. HIFU balloon catheter far away from ET probe (LAO projection); B. Direction of HIFU beam pointed away from ET probe (RAO projection); C. HIFU balloon catheter close to ET probe (LAO projection); D. Direction of HIFU beam pointed towards ET probe (RAO projection) (PN: phrenic nerve, SVC: superior caval vein, ET: esophageal temperature, HIFU: high-intensity focused ultrasound, balloon catheter: balloon catheter, RIPV: right inferior pulmonary vein, CS: coronary sinus, LAO: left anterior oblique, RAO: right anterior oblique)

The relative position from the balloon to the esophageal temperature probe is pulmonary vein and patient dependent. Selective pulmonary vein angiographies were performed to identify pulmonary vein ostia. Immediately before and after each ablation the relative position of the balloon to the esophageal temperature probe was fluoroscopically recorded in 30 degrees right anterior oblique (RAO) and 40 degrees left anterior oblique (LAO) simultaneously using a biplane fluoroscopy system (Figure 1).

To investigate the relationship between the esophageal temperature at the end of the ablation and the distance from the balloon to the esophageal temperature probe, we analyzed for each pulmonary vein ablation all fluoroscopic images. We compared images from ablation applications where the esophageal temperature at the end of the ablation was <38.5ºC or ≥38.5ºC, and <40.0ºC or ≥40.0ºC. The esophageal temperature cut-off of 38.5ºC has been recommended and used by other investigators,[6] and the temperature cut-off of 40.0ºC has been used by us to abort the ablation in order to prevent excessive heating of adjacent tissue. Because multiple temperature cut-offs are being used in clinical trials and because there is no consensus on which temperature cut-off best correlates with occurrence of adverse events, we chose to perform analyses on both 38.5ºC and 40.0ºC.

The distance measurement system was calibrated using the diameter of the 12F transseptal sheath. Since there was the possibility of a calibrating error, after calibration the known distance between the temperature sensors of the esophageal temperature probe was measured. Thus, the correct calibration could be confirmed or disproved.

In order to analyze distances in an easy and reproducable way, we have selected a number of easy to identify markers on the fluoroscopy screen.

**Figure 2:**
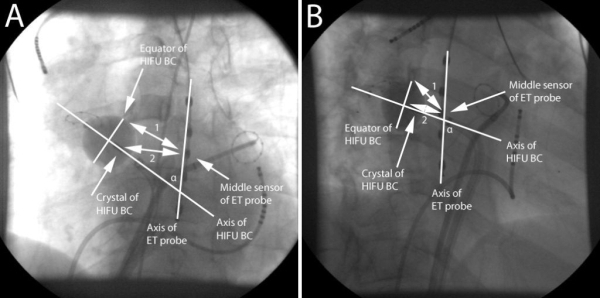
Analysis of distance from crystal and equator of HIFU balloon catheter to middle sensor of esophageal temperature probe and angle between axes of HIFU balloon catheter and esophageal temperature probe. A. LAO projection; B. RAO projection (HIFU: high-intensity focused ultrasound, balloon catheter: balloon catheter, LAO: left anterior oblique, 1: distance from equator of HIFU balloon catheter to middle sensor of ET probe, 2: distance from crystal of HIFU balloon catheter to middle sensor of ET probe, α: angle between axes of HIFU balloon catheter and ET probe, ET: esophageal temperature)

The analysis includes the distance from the crystal of the balloon to the middle sensor of the esophageal temperature probe; the distance from the equator of the balloon to the middle sensor of the esophageal temperature probe and the angle between the axes of the balloon and the esophageal temperature probe (Figure 2). Although the crystal is not the location where the tissue is being heated, it has a fixed distance to the sonicating ring of the balloon and is very easily visible using fluoroscopy. This makes the crystal a usable landmark. Also, the equator of the balloon is not the plane where the focus of the ultrasound beam is and where the tissue is being heated, this focus is located 2-6 mm distal from the equator. However, the plane of the focus of the ultrasound beam cannot be visualized, and it is the purpose of this analysis to use easy to recognize, fluoroscopically visible landmarks. Using these markers (crystal and equator) there is no need for additional post-hoc processing, since all measurements can be done in realtime.

All analyses were done for both the RAO and LAO projections. The use of these projections allows for reconstruction of a mental 3-dimensional image, in this way the virtual position and distance of one object or structure to another object or structure can be estimated. Since we aimed to provide a "realtime" analysis without need for additional post-hoc processing we have calculated all distances in the RAO and LAO projections separately, and did not calculate any distances using the combined orthogonal projections.

### Statistical analysis

Data mean ± standard deviation (SD) was used to describe continuous variables with normal distribution, otherwise median and range were used. For diagnostic parameters the absolute and relative frequencies were counted. A p value ≤0.05 was considered statistically significant. A receiver operator curve (ROC) analysis was performed to predict the minimal and maximal distances advised to stay below a cut-off esophageal temperature. The effect of distances and/or angles between the balloon and the esophageal temperature probe on the esophageal temperatures was assessed using a generalized mixed model under consideration of repeated measures taken of each patient. All statistical analyses (concerning mixed modelling) were performed with Statistical Analysis System (SAS) 9.2 using the MIXED and GLIMMIX procedures.

## Results

From July to December 2008 28 patients (18 male) with a mean age of 63±7 years were enrolled. The patient characteristics have been described in detail earlier.[4] At baseline, 2/28 patients (7%) used proton pump inhibitors, no other anti-acid drugs were used.

Endoscopy was performed in 26/28 patients (93%). Two out of 28 patients refused to undergo endoscopy but remained asymptomatic during follow-up. In 2/26 patients (8%) a small thermal lesion was found in the anterior part of the esophagus. Maximal esophageal temperatures were 37.3ºC and 41.9ºC, respectively. At repeat endoscopy after 2 weeks all thermal lesions were healed. No complaints occurred during follow-up.

One patient died because of an atrial-to-esophageal fistula after 31 days of follow-up. Maximal esophageal temperature was 39.1ºC.

In total 311 ablation applications were performed, from these 311 ablation applications the fluoroscopic recordings of 300 ablation applications (96%) were eligible for analysis. Because the recordings were done in both RAO and LAO projections, a total of 600 images were available for analysis. The fluoroscopic recordings of the remaining 11 ablation applications (4%) were not eligible for analysis, either because of failure to record the position of the balloon during the procedure or occurrence of a technical defect while attempting to retrieve the fluoroscopic recordings from the data storage medium.

### Relationship between esophageal temperature and position of the HIFU balloon catheter to esophageal temperature probe

**Figure 3:**
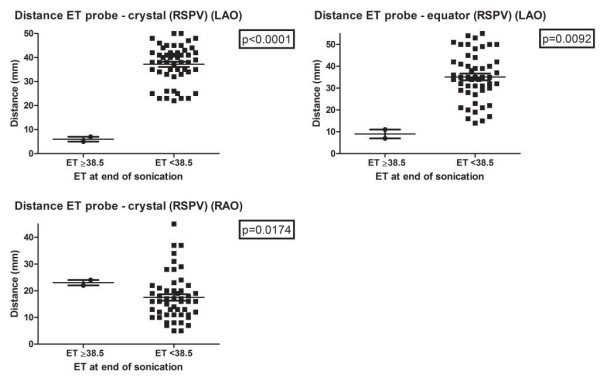
Relationship between esophageal temperature and position of HIFU balloon catheter to esophageal temperature probe during ablation of RSPV. Each black dot represents a high-intensity focused ultrasound (HIFU) ablation lesion. (ET: esophageal temperature probe temperature, RSPV: right superior pulmonary vein, RAO: right anterior oblique, LAO: left anterior oblique, mm: millimeter)

In the right superior pulmonary vein (RSPV) esophageal temperature increased to ≥38.5ºC when there was a significantly shorter distance from the crystal of the balloon to the esophageal temperature probe in LAO and from the equator of the balloon to the esophageal temperature probe in LAO. Interestingly, esophageal temperature also increased to ≥38.5ºC when there was a significantly longer distance from the crystal of the balloon to the esophageal temperature probe in RAO. This could be explained by the fact that in this position, when the distance is shorter (<20 mm), the focus of the HIFU beam is still located proximally from the esophagus and not in the proximity of the esophagus. However, when the distance is longer (>20 mm) the focus of the HIFU beam can reach the esophagus (Figure 3 and Table 1).

**Figure 4:**
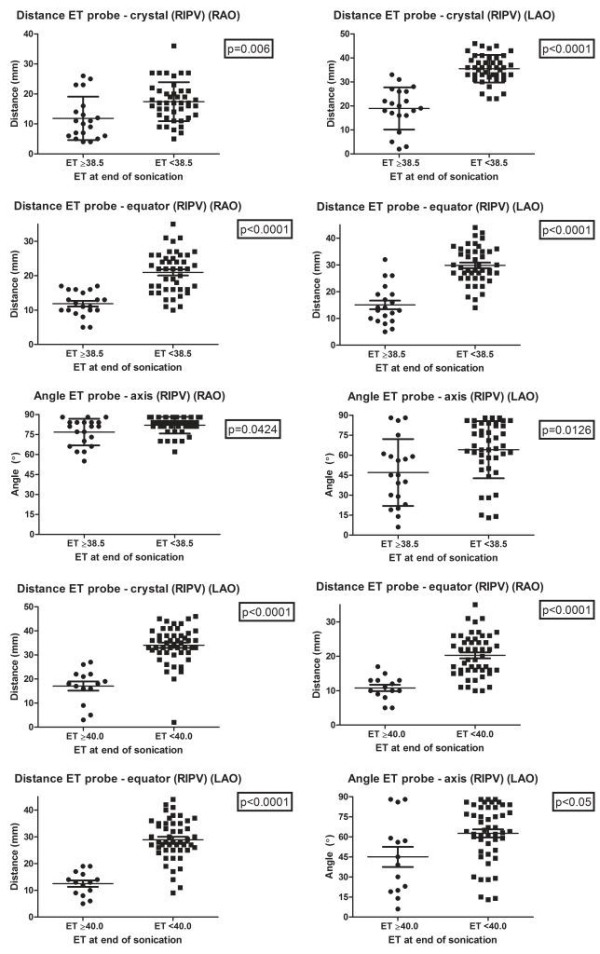
Relationship between esophageal temperature and position of HIFU balloon catheter to esophageal temperature probe during ablation of RIPV. Each black dot represents a high-intensity focused ultrasound (HIFU) ablation lesion. (ET: esophageal temperature probe temperature, RIPV: right inferior pulmonary vein, RAO: right anterior oblique, LAO: left anterior oblique, mm: millimeter)

In the right inferior pulmonary vein (RIPV) esophageal temperature increased to ≥38.5ºC when there was a significantly shorter distance from the crystal of the balloon to the esophageal temperature probe in both RAO and LAO and from the equator of the balloon to the esophageal temperature probe in both RAO and LAO. Also esophageal temperature increased to ≥38.5ºC when there was a significantly smaller angle between the axes of the balloon and the esophageal temperature probe in both RAO and LAO (Figure 4 and Table 1).

**Figure 5:**
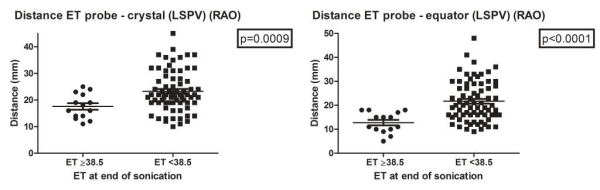
Relationship between esophageal temperature and position of HIFU balloon catheter to esophageal temperature probe during ablation of the LSPV. Each black dot represents a high-intensity focused ultrasound (HIFU) ablation lesion. (ET: esophageal temperature probe temperature, LSPV: left superior pulmonary vein, RAO: right anterior oblique, LAO: left anterior oblique, mm: millimeter)

**Figure 6:**
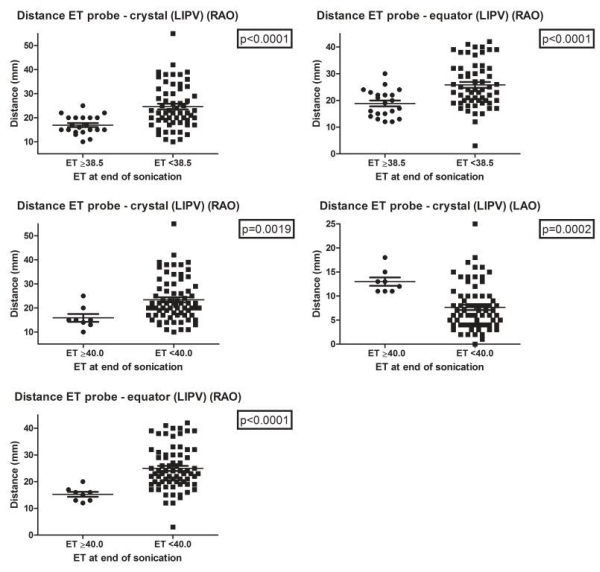
Relationship between esophageal temperature and position of HIFU balloon catheter to esophageal temperature probe during ablation of LIPV. Each black dot represents a high-intensity focused ultrasound (HIFU) ablation lesion. (ET: esophageal temperature probe temperature, LIPV: left inferior pulmonary vein, RAO: right anterior oblique, LAO: left anterior oblique, mm: millimeter)

In the left superior pulmonary vein (LSPV) and the left inferior pulmonary vein (LIPV) esophageal temperature increased to ≥38.5ºC when there was a significantly shorter distance from the crystal of the balloon to the esophageal temperature probe in RAO and from the equator of the balloon to the esophageal temperature probe in RAO (Figure 5,6 and Table 1).

In the RIPV esophageal temperature increased to ≥40.0ºC when there was a significantly shorter distance from the crystal of the balloon to the esophageal temperature probe in LAO and from the equator of the balloon to the esophageal temperature probe in both RAO and LAO. Also esophageal temperature increased to ≥38.5ºC when there was a significantly smaller angle between the axes of the balloon and the esophageal temperature probe in LAO (Figure 4 and Table 1).

In the LSPVs where esophageal temperature increased to ≥40.0ºC there were no significant differences between the distances from the crystal or the equator of the balloon to the esophageal temperature probe or the angle between the axes of the balloon and the esophageal temperature probe as compared to LSPVs where esophageal temperature increased to <40.0ºC.

In the LIPV esophageal temperature increased to ≥40.0ºC when there was a significantly shorter distance from the crystal of the balloon to the esophageal temperature probe in RAO and from the equator of the balloon to the esophageal temperature probe in RAO. Interestingly, esophageal temperature also increased to ≥40.0ºC when there was a significantly longer distance from the crystal of the balloon to the esophageal temperature probe in LAO. (Figure 6 and Table 1).

### Relationship between esophageal temperature and location of pulmonary vein

During HIFU application maximal esophageal temperature differed greatly per pulmonary vein, with lowest maximal esophageal temperature during ablation of the RSPV (p<0.0001). Mean maximal esophageal temperature ± SD [range] in the LSPV was 37.3±1.0ºC [35.9-41.2], in the LIPV 37.8±1.4ºC [36.2-42.2], in the RSPV 37.0±0.6ºC [36.2-39.8] and in the RIPV 38.2±1.9ºC [36.2-44.0]. During a first HIFU ablation application in the RIPV the pulmonary vein was isolated after 2 seconds and the change in temperature was 8.2ºC, despite use of Power Modulation. Due to the excessive change in temperature the application was aborted. The pulmonary vein remained isolated. Also, changes in esophageal temperature differed significantly between the different pulmonary veins, with the least changes in esophageal temperature during ablation of the RSPV (p<0.0001).

### Relationship between esophageal injury and position of the HIFU balloon catheter to esophageal temperature probe

In 3 patients esophageal injury could be demonstrated: 2 patients had small thermal lesions found during endoscopy and 1 patient died from a lethal atrial-to-esophageal fistula.

In the first patient with a small thermal lesion in the esophagus the maximal esophageal temperature was 37.3ºC. The distance between the balloon and the esophageal temperature probe was always relatively long, compared to other ablations in the respective pulmonary vein.

In the second patient with a small thermal lesion in the esophagus the maximal esophageal temperature during ablation of the LIPV was 41.9ºC. Here, the distance between the crystal of the balloon and the esophageal temperature probe in the RAO view was relatively short, as was the distance between the equator of the balloon and the esophageal temperature probe in the RAO view. As expected, the distance between the crystal of the balloon and the esophageal temperature probe in the LAO view was relatively long.

The patient who suffered from the lethal atrial-to-esophageal fistula had a maximal esophageal temperature of 39.1ºC when ablation of the LSPV was performed. Here, the distance between the crystal of the balloon and the esophageal temperature probe in the RAO view was relatively short, as was the distance between the equator of the balloon and the esophageal temperature probe in the RAO view.

In these 3 patients with demonstrated esophageal damage ablation times needed to isolate the respective pulmonary veins were similar to those of patients without demonstrated esophageal damage.

### Relationship between esophageal temperature and success of pulmonary vein isolation

The mean maximal esophageal temperature observed during a total of 311 HIFU ablation applications was 37.6±1.3ºC with a mean change in esophageal temperature of 0.8±0.6ºC. Esophageal temperature increased to ≥38.5ºC in 60 (19%) and to ≥40ºC in 26 (8%) of all ablation applications. Nine pulmonary veins (RIPV 5, LIPV 3 and LSPV 1) could not be isolated with use of HIFU due to excessive esophageal heating.

## Discussion

Esophageal injury with atrial-to-esophageal fistula formation was initially described after left atrial ablation with radiofrequency current (RFC) and HIFU catheter ablation.[3,6] More recently, reports on esophageal injury after pulmonary vein isolation with balloon systems using cryothermal energy and an endoscopic laser ablation system have been published.[7,8]

Unfortunately, no defined strategy has been identified to avoid esophageal injury during catheter ablation. Although realtime monitoring of esophageal temperature is feasible during the catheter ablation procedure for atrial fibrillation, recent experimental studies have demonstrated that esophageal injury can occur without any detected increase in temperature measured inside the esophageal lumen.[9,10 ]

Also, recent clinical studies reported to have found no correlation between esophageal temperature and occurrence of esophageal ulcers. Cummings et al reported significant differences between luminal esophageal temperature and external esophageal tissue temperature during RFC catheter ablation of the left atrium.[6,11] When the injury comes from the outside of the esophagus, where temperature is highest, this might explain why ulcers can develop in patients where luminal esophageal temperature does not increase significantly. However, at the present time there is no better alternative for monitoring the condition of the esophagus.

We conducted an analysis to investigate the correlation between the relative position of the balloon to the esophageal temperature probe and the changes in esophageal temperature. This study is the first to systematically evaluate not only the variable position of the esophagus in relation to the balloon, but also changes in esophageal temperature during ablation with HIFU.

There was a clear relationship between distance and/or angle of the balloon to the esophageal esophageal temperature probe and maximal esophageal temperature at the end of the ablation. In general, shorter distances and smaller angles caused higher esophageal temperatures. These results are in concordance with the study of Okumura et al. They concluded that direct rapid mechanical heating and subsequent conductive heating effects of HIFU as the mechanism for phrenic nerve injury occur within 4 to 7 mm from the balloon surface.[12]

The changes in temperature per pulmonary vein differ too: the change in temperature was highest in the RIPV and LIPV, whereas the RSPV had the least change in temperature. The anatomical relationship of the esophagus and the pulmonary vein plays a very important role, as was demonstrated by Tsao et al.[13] They found that there are 2 major types of esophageal routes along the posterior left atrium: 88% of patients had the esophagus running with the lower portion of the esophagus close to the ostium of the LIPV (type 1), whereas only 12% of patients had the esophagus running with the lower portion of the esophagus close to the ostium of the RIPV (type 2). The mean shortest distance from the esophagus to the RSPV in their type 1 patients (88% of the total) was 28.4±6.1 mm. This could very well explain our finding that the esophageal temperature increased to ≥38.5ºC when there was a significantly longer distance from the crystal of the balloon to the esophageal temperature probe in RAO. When the distance is shorter (<20 mm), the focus of the HIFU beam is located proximally from the esophagus and not in the proximity of the esophagus. However, when the distance is longer (>20 mm) the exit of the HIFU beam is still proximal from the esophagus and the focus of the HIFU beam can reach the esophagus.

The same holds true for the LIPV, Tsao et al found that the mean shortest distance from the esophagus to the LIPV in their type 1 patients (88% of total) was 2.8±2.5 mm. We found that esophageal temperature also increased to ≥40.0ºC when there was a significantly longer distance from the crystal of the balloon to the esophageal temperature probe in LAO. This could be explained by the fact that in this position, when the distance is very short, the focus of the HIFU beam is already located distally from the esophagus and thus the HIFU beam is directed away from the esophagus. However, when the distance is longer the exit of the HIFU beam is still proximal from the esophagus and the focus of the HIFU beam can reach the esophagus.

## Limitations

1. The number of esophageal lesions found in this study is too small to predict occurrence of esophageal lesions based on distance and/or angle of balloon to the esophageal temperature probe.

2. Differences in size of the left atrium, differences in size and shape of the pulmonary vein ostium, patient size, position of the esophagus in relation to the left atrium and position of the esophageal temperature probe in relation to the anterior esophageal wall can influence the outcome of the measurements.

3. Although the relative position of the balloon to the esophageal temperature probe was fluoroscopically recorded immediately before and after each ablation and post-hoc analysis showed that there was no change in distance between the two fluoroscopic recordings, we cannot guarantee that the distances stayed constant between the two fluoroscopic recordings. But, because the distances post-ablation were always similar to the distances pre-ablation one could assume that there has been no significant change in position of the balloon during the ablation.

## Conclusions

There is a relationship between the distance and/or the angle of the HIFU balloon to the esophageal temperature probe and esophageal temperature. For most fluoroscopic projections counts: the shorter the distance, the higher the esophageal temperature will become.

## Figures and Tables

**Table 1:**
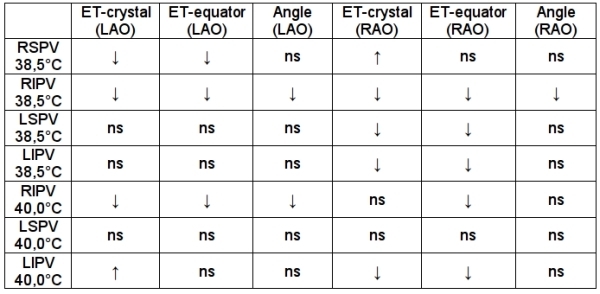
Overview per pulmonary vein and cut-off esophageal temperature of significant differences in distance or angle between the HIFU balloon catheter and the esophageal temperature probe when esophageal temperature reaches the cut-off value

RSPV: right superior pulmonary vein, RIPV: right inferior pulmonary vein, LSPV: left superior pulmonary vein, LIPV: left inferior pulmonary vein, LAO: left anterior oblique, RAO: right anterior oblique, ET: esophageal temperature, ↓: significant shorter distance or smaller angle when ET is above cut-off ET, ↑: significant longer distance when ET is above cut-off ET, ns: not significant
